# Numerical and Experimental Evaluation of Winding-Corner Integrity in an Fe–5.0 wt.%Si Soft Magnetic Composite Stator Core for Direct-Winding Applications

**DOI:** 10.3390/ma19183876

**Published:** 2026-09-11

**Authors:** Minseop Sim, Seonbong Lee

**Affiliations:** 1 Department of Mechanical Engineering, Keimyung University, Daegu 42601, Republic of Korea; 5587911@stu.kmu.ac.kr; 2 Department of Automotive Engineering, Keimyung University, Daegu 42601, Republic of Korea

**Keywords:** soft magnetic composite (SMC), Fe–Si alloy, stator core, direct winding, winding corner, wire tension, corner loading, finite element analysis, mechanical integrity, optical inspection

## Abstract

Direct winding places magnet wires in direct contact with soft magnetic composite (SMC) stator cores, making the winding-corner response important for component design. This study evaluated an Fe–5.0 wt.%Si SMC stator core through finite element analysis and corner-loading tests. Based on a wire tension of 1.44 N/turn and a 90° change in winding direction, the resultant load was 2.04 N per turn and 38.7 N for 19 turns. An engineering verification load of 177 N was defined from the mean nominal 0.2% proof load of the AI-EIW wire. The equivalent distributed model at 38.7 N and the representative one-turn circular-contact model at 2.04 N predicted elastic responses without effective strain. At 177 N, the maximum effective stress and strain were 876.9 MPa and 0.0132, and the maximum resultant displacement after unloading was 0.85 μm. Component-level tests showed continuous force increases to the prescribed loads. Optical microscopy at 20× magnification identified no indentation, cracking, edge chipping, particle detachment, or corner-profile change after unloading, although the FEA-predicted sub-micrometer localized deformation was not directly quantified by the optical evaluation. These results characterize the local mechanical response under the defined reference winding and engineering verification conditions.

## 1. Introduction

Permanent-magnet synchronous motors (PMSMs) are widely used in automotive traction systems, industrial actuators, and high-efficiency rotating machinery owing to their high power density, high efficiency, and excellent torque characteristics [1]. PMSMs can be classified into various configurations according to the direction of magnetic-flux propagation and the arrangement of the permanent magnets [2,3,4]. A radial-flux permanent-magnet (RFPM) motor is a representative configuration in which the magnetic flux is formed in the radial direction perpendicular to the axis of rotation [5,6]. SMCs are powder-metallurgy-based soft magnetic materials manufactured by forming an electrically insulating layer on the surfaces of magnetic powder particles, followed by compaction [7]. The insulating layers formed between individual particles restrict eddy-current circulation paths, while the powder compaction process is advantageous for manufacturing cores with complex three-dimensional geometries and multidirectional magnetic-flux paths [7]. Fe–Si-based SMCs have been investigated as core materials for motors, inductors, and electromagnetic actuating components because of their relatively high saturation magnetization and electrical resistivity [8]. Furthermore, their ability to form geometries with segmented teeth, curved surfaces, and localized cross-sectional variations can increase the design flexibility of complex stator structures [9,10].

The final properties of SMCs depend on the powder composition, particle-size distribution, insulation treatment, lubricant content, compaction conditions, heat-treatment conditions, and final density [11,12,13]. These manufacturing variables affect not only permeability and core loss but also compressive strength, hardness, and resistance to localized loading [14]. Because powder compacts have heterogeneous structures containing interparticle bonding regions, residual pores, and insulating layers, differences may arise between the average mechanical properties measured using bulk specimens and the local deformation of actual components, depending on their geometry and contact conditions [15]. In particular, loads are transferred through a limited contact area at regions with a small corner radius, potentially resulting in localized indentation, particle detachment, corner chipping, and microcracking.

The stator core investigated in this study is a segmented SMC structure used in a radial-flux surface-mounted permanent-magnet synchronous motor (RF-SPMSM) and employs a winding method in which magnet wire is wound directly onto the stator core [16,17]. The stator structure for an axial-flux permanent-magnet (AFPM) motor examined in our previous work had a relatively simple geometry, allowing the coil to be wound externally using a separate fixture and subsequently inserted into the stator core [18]. In contrast, the RF-SPMSM stator core investigated in the present study has a complex geometry comprising slot openings, teeth, and connecting sections and therefore employs direct winding, in which the wire is wound along the slots and corners of the actual core.

After winding, the coil covers the interior of the slots and the corners of the stator core, making direct observation of the surface condition in these regions difficult. Once the motor assembly has been completed, optical access to the internal winding corners is limited, and separating the core or removing the coil may alter the original contact and deformation states. Therefore, a procedure is required to evaluate the deformation and surface condition of the stator-core winding corners under the reference winding condition and under a higher verification condition defined within the mechanical deformation limit of the wire.

Conventional evaluations of the mechanical properties of SMCs have primarily focused on the compressive strength, flexural strength, and hardness of cylindrical or rectangular bulk specimens [19,20,21]. These properties provide criteria for assessing the intrinsic strength of the material and the reproducibility of its manufacturing process [22,23,24,25,26]. In an actual stator core, however, the slot and tooth geometries, corner radius, loading direction, contact area, and support conditions determine the local stress and displacement distributions [27]. Accordingly, numerical analysis and verification testing that reflect the actual component geometry are required in conjunction with material-level mechanical characterization [27,28,29]. However, studies directly evaluating the winding-induced mechanical response of a fully manufactured SMC stator core have not been reported, particularly those applying loads derived from wire tension to the winding corner and correlating the numerical results with the component-level load–displacement response and surface condition before and after loading.

Furthermore, the maximum or fracture load of the wire exceeds the load range preceding permanent deformation and is therefore not representative of a practical verification condition for winding. Accordingly, the nominal 0.2% proof load was adopted as a higher engineering verification load, representing the onset of permanent deformation of the wire, to evaluate the response of the stator-core winding corner under a loading condition beyond the reference winding load.

Accordingly, this study comprehensively evaluated the material-level mechanical properties of an Fe–Si SMC and the deformation of the winding corners of a stator core. First, the compressive strength and Vickers hardness were measured using separate specimens fabricated from the same Fe–Si SMC powder system. The actual winding condition was established based on the recommended winding tension for the diameter of the AI-EIW wire used and the number of turns per slot, while a high-load verification condition was determined based on the nominal 0.2% proof load of the wire measured through a standardized tensile test.

This study integrates the mechanical properties of the bulk SMC material, the tensile properties of the AI-EIW wire, finite element analysis reflecting the actual stator-core geometry, and component-level corner loading tests. Through this integrated approach, a method is proposed for evaluating the winding load applied to a complex-shaped SMC stator core for an RF-SPMSM employing direct winding. The deformation and surface damage of the winding corners were quantitatively evaluated over the range extending from the actual winding condition to a high-load verification condition based on the nominal 0.2% proof load of the wire.

## 2. Materials and Methods

### 2.1. Fe–Si SMC Stator-Core Specimen and Direct-Winding Evaluation Region

The segmented stator core for the RF-SPMSM investigated in this study was fabricated using FF50SP Fe–5.0 wt.%Si pre-alloyed powder. The raw powder consisted of 94.85 wt.% Fe, 5.02 wt.% Si, and 0.13 wt.% O, as presented in Table 1. Based on the mass of the base powder, 0.25 wt.% H_3_PO_4_, 0.25 wt.% polyimide, 1.0 wt.% MoS_2_, and 0.3 wt.% graphite were added [30]. The particle-size distribution of the powder and the detailed mixing and insulation-treatment procedures were reported in our previous study [30,31,32].

The stator core was fabricated using the 50–100% 2P1A (2-Pressing-1-Annealing) condition established in our previous study [32]. The first pressing was performed at 350 °C to a first-pressing level of 50%, followed by the second pressing at 550 °C under an Ar atmosphere to a final pressing level of 100% [32]. The compact was subsequently heat-treated at 720 °C for 30 min under an N_2_–10% H_2_ mixed-gas atmosphere. The SMC cores fabricated using this process were used for the winding-corner evaluation.

**Figure 1:**
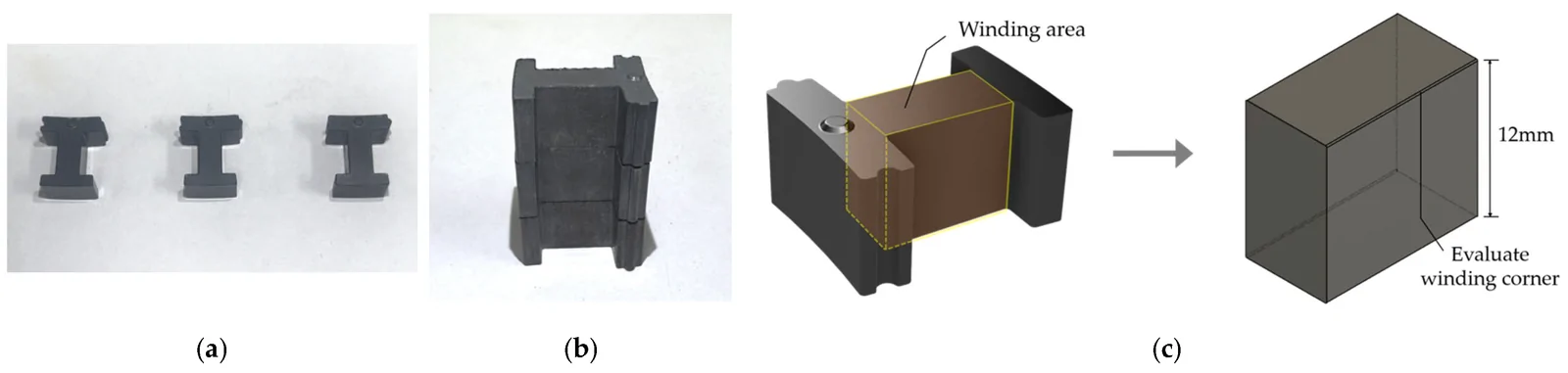
Fe–Si SMC stator-core configuration and direct-winding evaluation region: (**a**) individual 12 mm cores, (**b**) a 36 mm segment comprising three axially stacked cores, and (**c**) the direct-winding region and evaluated winding corner.

The fabricated core has a segmented structure assembled into the stator, with the slot and tooth formed as a single compact. The nominal axial height of each SMC core was 12 mm, and three identically shaped cores were stacked in the axial direction to form a stator-core segment with a total height of 36 mm. The target bulk density was 7.35 g/cm^3^. Figure 1a,b show three individual SMC cores with an axial height of 12 mm and the 36 mm stator-core segment formed by stacking these cores in the axial direction, respectively [30].

The fabricated segmented SMC stator core employs a configuration in which the wire is wound directly around the tooth. During winding, the wire travels around the tooth and contacts the corner at which its direction changes, generating a contact load at the corner owing to the winding tension. Figure 1c shows the direct-winding region defined on a single SMC core. Among the corners within the winding region, the corner at which the direction of wire travel changes was selected as the target of the mechanical evaluation and is hereafter referred to as the winding corner. This region was designed with an angle of 90°. For corners without a separately specified radius in the core design, a nominal corner radius of 0.1 mm was assigned; accordingly, the winding corner evaluated in this study was defined with a radius of 0.1 mm. The subsequent finite element analysis, corner-loading tests, and optical microscopy observations were conducted on the same winding corner.

### 2.2. Bulk Mechanical Properties of the Fe–Si SMC

The compressive strength and Vickers hardness were measured to characterize the basic mechanical properties of the Fe–5.0 wt.%Si SMC fabricated using the 2P1A process [32]. Because the mechanical properties of powder compacts are affected not only by the powder composition but also by the pressing conditions and heat-treatment history, the bulk compressive resistance and local indentation resistance of the stator core after the complete manufacturing process were quantitatively evaluated [33,34]. Compressive strength was defined as the maximum compressive stress sustained by the compact under axial loading, whereas Vickers hardness represented the local resistance of the surface to indentation. These properties were used as reference values representing the initial mechanical state of the SMC core evaluated in the subsequent winding-corner loading analysis.

Five cylindrical specimens with a diameter of 5.2 mm were used for the compression tests. The tests were performed using an INSTRON 5569 universal testing machine (Instron Corporation, Norwood, MA, USA) at a crosshead speed of 1 mm/min. The compressive strength of each specimen was calculated by dividing the measured maximum compressive load by its initial cross-sectional area. Vickers hardness was measured at five locations under an HV1 test condition in accordance with KS B 0811:2003 [35,36].

The Fe–5.0 wt.%Si SMC exhibited a compressive strength of 1226.4 ± 22.0 MPa, with a coefficient of variation (CV) of 1.79%. The Vickers hardness was 358.2 ± 6.1 HV1, with a CV of 1.70%. These results demonstrate that the SMC fabricated using the 2P1A process exhibited consistent compressive strength and surface hardness across the repeated measurements. The subsequent winding-corner analysis and component-level tests were therefore conducted on Fe–5.0 wt.%Si SMC cores with these mechanical properties. The test conditions and measured results are summarized in Table 2.

### 2.3. AI-EIW Wire Specification and Equivalent Reference Winding Load

An AI-EIW round magnet wire with a nominal conductor diameter of 1.20 mm was used for the stator winding, corresponding to a nominal conductor cross-sectional area of 1.131 mm^2^. According to the winding-tension data provided by LS Cable & System, the recommended winding tension for an AI-EIW wire with a nominal conductor diameter of 1.20 mm is 147 gf, equivalent to 1.44 N per turn in SI units [37].

**Figure 2:**
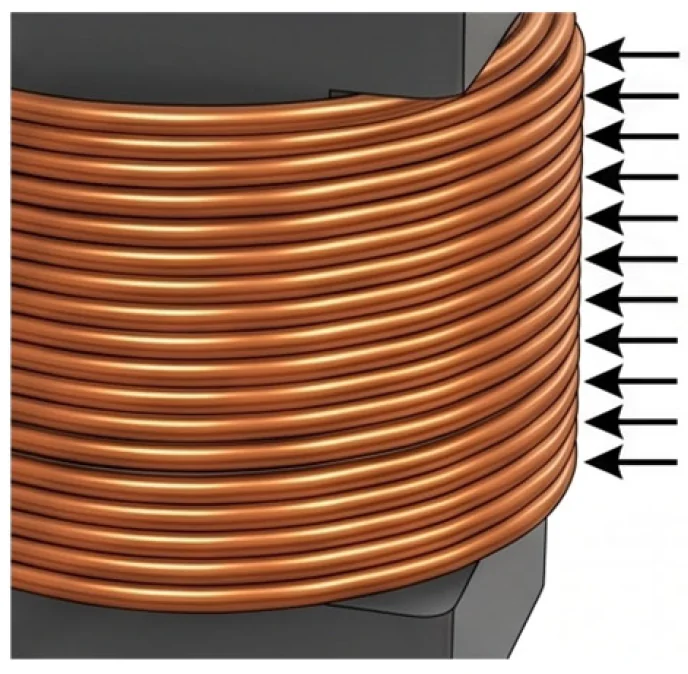
Schematic illustration of the AI-EIW wire arrangement and load-transfer direction at the winding corner. The arrows represent the direction of the transferred winding load.

During winding, the wire is placed around the tooth and undergoes a nominal 90° change in direction as it passes over the winding corner. The two wire segments adjacent to the corner were assumed to carry the same recommended winding tension, *Tr*. Because the two tension directions are perpendicular, the resultant load transferred to the corner by one turn is $\sqrt{2}$*Tr* and acts along the bisector of the 90° corner. Figure 2 schematically illustrates the wire arrangement and load-transfer direction at the winding corner. The wires depicted in the figure illustrate the load-transfer configuration, while the actual winding specification was 19 turns per slot. The total resultant load acting on a single winding corner was calculated as follows:

$$R_{turn}=2T_{r}sin\left(\frac{90{}^{\circ}}{2}\right)=\sqrt{2}T_{r}$$

(1) $$F_{r}=nR_{turn}=19\sqrt{2}\left(1.44\right)=38.69\approx 38.7\;N$$

where *n* is the number of wire turns passing over the same winding corner, and *Tr* is the manufacturer-recommended winding tension per turn. Accordingly, *Fr* = 38.7 N was defined as the vector-resolved Equivalent Reference winding Load (ERL) acting on a single winding corner and was applied in the subsequent finite element analysis. This value represents a nominal tension-transfer condition. Wire–core friction, contact conditions, tension variation, and the winding path may affect the local load transferred during the actual winding process.

### 2.4. AI-EIW Wire Tensile Test and High-Load Verification Load

The tensile properties of the AI-EIW wire were measured to establish a higher verification condition for evaluating the behavior of the winding corner beyond the ERL defined in Section 2.3. An AI-EIW wire with a nominal conductor diameter of 1.20 mm and a nominal conductor cross-sectional area of 1.131 mm^2^ was used. The tensile tests were conducted in accordance with KS C IEC 60851-3 using an initial free length of 200 mm and a crosshead speed of 5 mm/s. A total of five wire specimens were tested [38,39].

**Figure 3:**
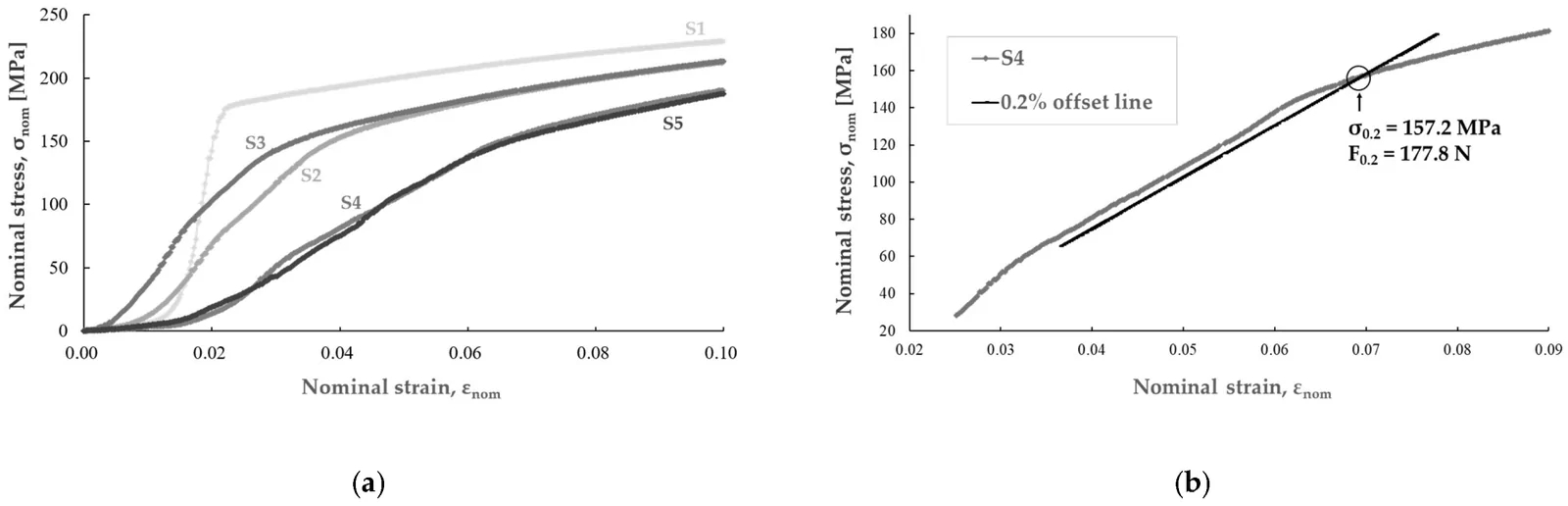
Nominal stress–strain responses of the five AI-EIW wire specimens: (**a**) comparison of specimens S1–S5 and (**b**) representative determination of the nominal 0.2% proof stress and corresponding proof load for specimen S4.

The nominal stress–strain curve of each specimen was obtained from the measured load and crosshead displacement. The nominal stress was calculated by dividing the measured load by the initial conductor cross-sectional area of 1.131 mm^2^, and the nominal strain was calculated by dividing the crosshead displacement by the initial free length of 200 mm. The nominal stress–strain responses of the five specimens are presented in Figure 3a.

The 0.2% offset method was applied to each specimen to quantitatively determine the proof load used for this higher verification condition. A linear regression was performed over the nominal stress range of 80–140 MPa to determine the reference slope, and a line retaining this slope was shifted by 0.002 along the strain axis [40]. The nominal 0.2% proof stress, *σ*_0.2_, was determined from the intersection between the shifted line and the measured nominal stress–strain curve, and the corresponding 0.2% proof load, *F*_0.2_, was then calculated. Figure 3b presents specimen S4 as a representative example because its proof load was closest to the mean value of the five specimens. The *σ*_0.2_ and *F*_0.2_ values of S4 were 157.2 MPa and 177.8 N, respectively.

The *F*_0.2_ values of specimens S1–S5 were 201.14, 172.65, 167.11, 177.84, and 166.03 N, respectively, resulting in a mean value of 176.95 ± 14.32 N. The corresponding mean nominal 0.2% proof stress was 156.47 ± 12.66 MPa. The individual and mean values are summarized in Table 3.

Based on the mean 0.2% proof load obtained from the five specimens, *Fu* = 177 N was established as the High-load Verification Load (HVL). The HVL was adopted to provide an engineering safety margin beyond the ERL for component-level verification. *Fu* does not represent the wire tension applied during the actual winding process or the summed winding load of the 19 turns; rather, it is a component-level verification load established from the nominal 0.2% proof load of the AI-EIW wire itself. Accordingly, the ERL and HVL were applied in the subsequent finite element analysis and winding-corner loading tests.

## 3. Simulation

### 3.1. Finite Element Analysis of the Winding Corner

A three-dimensional finite element analysis was performed using DEFORM-3D v14.0 to evaluate the local deformation and stress distribution at the stator-core winding corner under the loads transferred by the AI-EIW wire. The analysis focused on the 90° winding corner of the stator core, which is in direct contact with the wire. A corner radius of 0.1 mm was applied to reproduce the actual core geometry. Two loading representations were considered: an equivalent distributed model representing the total resultant load transferred by the 19 winding turns and a representative one-turn circular-contact model used to examine the local response associated with the round-wire geometry.

The Fe–5.0 wt.%Si SMC core after pressing and heat treatment was modeled as a homogeneous, isotropic elastoplastic material to represent the macroscopic mechanical response of the fully processed core at the component scale [41,42]. A continuum elastoplastic approach has previously been applied to describe the macroscopic mechanical behavior of a bulk material manufactured by powder metallurgy [41]. The material properties were defined based on separate uniaxial compression tests of cylindrical specimens fabricated from the same Fe–5.0 wt.%Si powder system under the same pressing and heat-treatment conditions as the stator core [43]. An effective elastic modulus of 14.042 GPa and the post-yield flow-stress data as a function of effective plastic strain were assigned to the core material. A Poisson’s ratio of 0.28 was assigned to the core material. The loading bodies used in the two models were modeled as rigid bodies.

**Figure 4:**
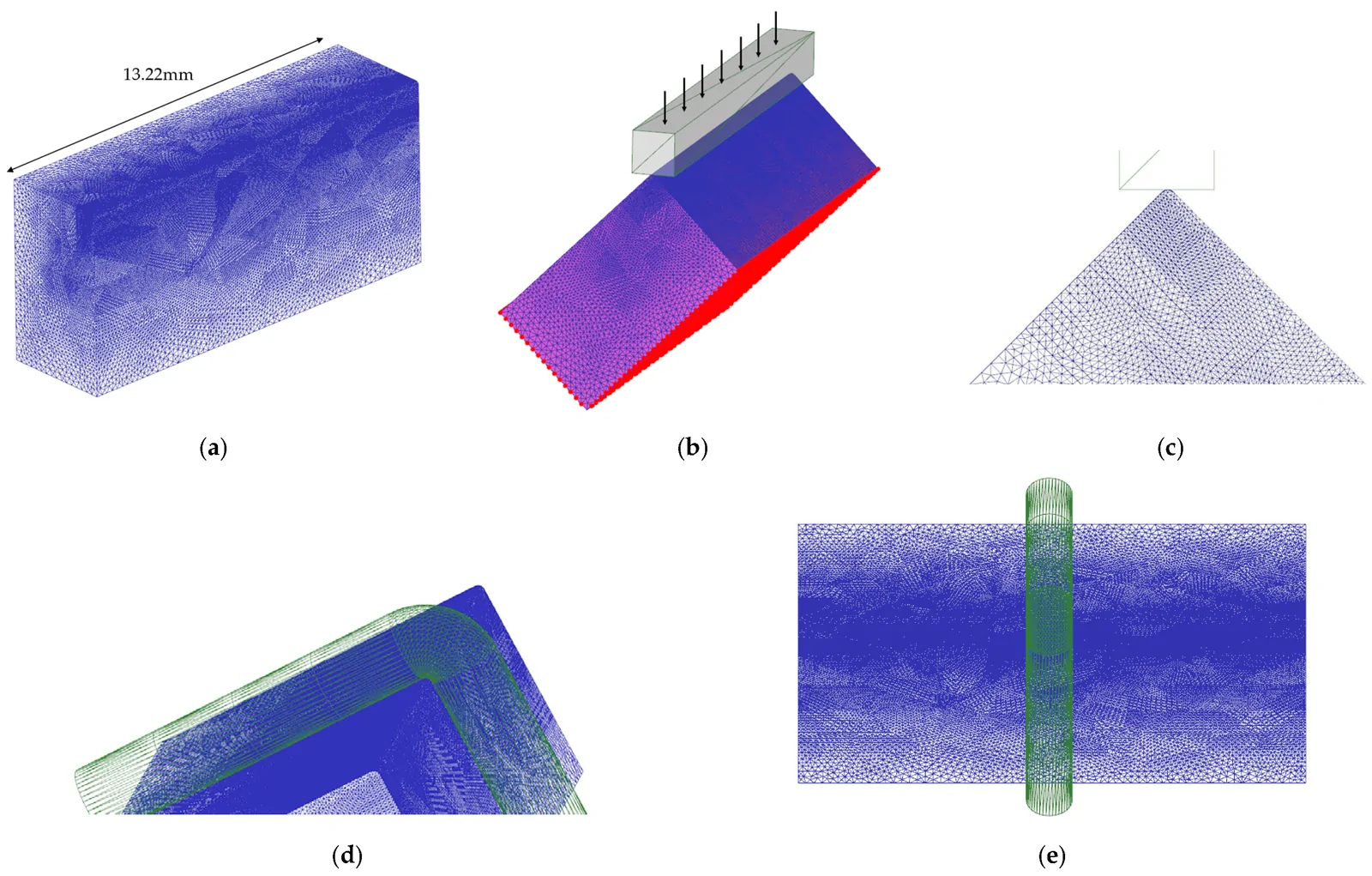
Finite element models and boundary conditions for the winding-corner analysis. Equivalent distributed model: (**a**) one-quarter reduced model of the winding region, (**b**) loading configuration and constrained support region, where the arrows indicate the direction of the applied loading, and (**c**) locally refined mesh around the winding corner. Representative one-turn circular-contact model at $R_{turn}$ = 2.04 N: (**d**) round-wire contact at the winding corner and locally refined contact mesh and (**e**) top view of the contact configuration.

Using the entire stator core as the analysis domain would distribute a large number of elements in regions remote from the winding corner of interest. Therefore, a local analysis domain was constructed based on the winding region shown in Figure 1c. Because rounded corners with identical geometries are repeated within this region, a one-quarter reduced model was generated by dividing the winding region in the longitudinal and transverse directions while retaining one rounded corner as the evaluation region. This approach restricted the analysis domain to the vicinity of the winding corner and enabled intensive mesh refinement at the small-radius contact region beneath the loading body. The overall mesh of the reduced model is shown in Figure 4a.

In the equivalent distributed model, the upper loading body was configured as a rectangular rigid body with a cross section of 1.2 × 1.2 mm^2^ and a length of 13.22 mm along the longitudinal direction of the winding corner. The total resultant load transferred by the 19 winding turns was homogenized over the 13.22 mm winding-corner length through the equivalent loading body. The core was oriented such that the bisector of the evaluated corner was aligned with the vertical movement direction of the loading tool. To reproduce the support condition of the actual corner-loading test, in which the core was held by a fixture at an inclination of approximately 45°, displacements in the X, Y, and Z directions were constrained over the lower core region in contact with the fixture. The equivalent loading configuration and constrained support region are shown in Figure 4b.

Local mesh refinement was applied around the winding corner to accurately represent its small curvature and the contact region between the loading body and the core. The relative element size in the refined region was set to 0.25 of the global element size, and the minimum element size at the corner contact region was 0.0455 mm. The refined mesh following the corner curvature and its transition to the surrounding mesh are shown in Figure 4c.

A representative one-turn circular-contact model was constructed to examine the local response of the SMC core associated with the nominal cross-sectional geometry of the AI-EIW wire. A rigid round-wire-shaped loading body with a diameter of 1.20 mm was arranged along the 90° winding path around the core corner with a radius of 0.1 mm and positioned at the midpoint of the 13.22 mm winding-corner length. The same core material properties and support constraints as those of the equivalent distributed model were applied. A close-up of the locally refined contact mesh in the representative one-turn circular-contact model is shown in Figure 4d.

A friction coefficient of 0.1 was applied to the contact interface between the rigid loading body and the SMC core in both models [28] ]. In the equivalent distributed model, the upper loading body was displaced in the −Z direction. Loading was terminated when the vertical reaction force reached either the ERL or the HVL, followed by unloading. In the representative one-turn model, the round-wire-shaped loading body was displaced along the corner bisector until the reaction force reached $R_{turn}$ = 2.04 N, corresponding to the resultant of the recommended wire tension of 1.44 N over a 90° change in the winding direction. The contact condition allowed separation between the loading tool and the winding corner during unloading. For each loading condition, the reaction force–displacement response and the distributions of effective stress and effective strain at the winding corner were evaluated. The resultant displacement and Z-direction displacement were also examined after unloading.

### 3.2. Numerical Results of the Winding-Corner Analysis

**Figure 5:**
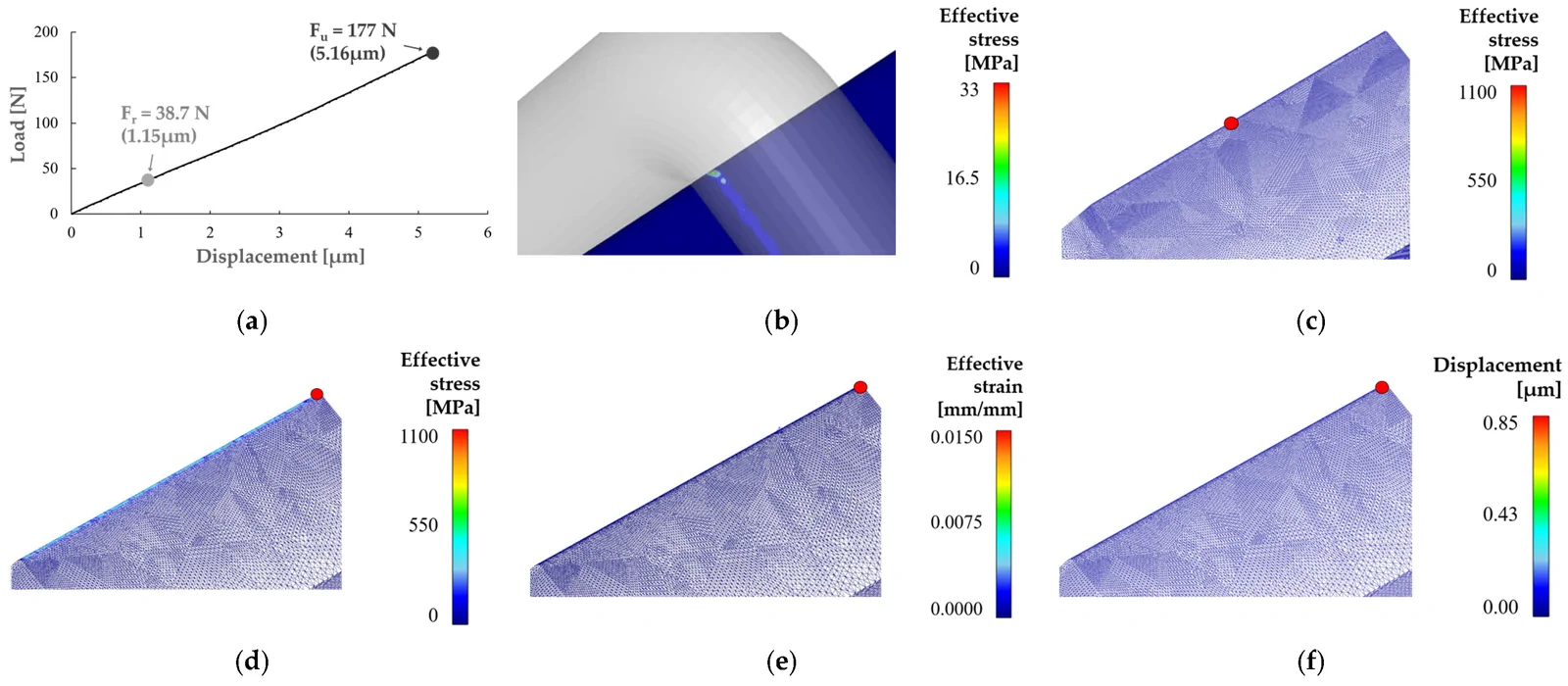
Numerical response of the SMC winding corner under the applied loads: (**a**) reaction force–loading-body displacement response of the equivalent distributed model, (**b**) effective-stress distribution in the representative one-turn circular-contact model at $R_{turn}$ = 2.04 N, (**c**) effective-stress distribution in the equivalent distributed model at *Fr* = 38.7 N (ERL), (**d**) effective-stress distribution at *Fu* = 177 N (HVL), (**e**) effective-strain distribution at *Fu* = 177N (HVL), and (**f**) resultant-displacement distribution after unloading from *Fu*. An independent stress scale was used in (**b**) to display the localized stress distribution under the per-turn load. The red circles indicate the locations of the maximum values in the corresponding contour plots.

Figure 5a shows the relationship between the loading-body displacement and reaction force in the equivalent distributed model. The reaction force increased continuously with increasing loading-body displacement. The ERL was reached at a displacement of 1.15 μm, whereas the HVL was reached at 5.16 μm.

In the representative one-turn circular-contact model, the per-turn resultant load of $R_{turn}$ = 2.04 N was reached at a loading-body displacement of 1.69 μm. The maximum effective stress in the SMC core was 22.9 MPa and was localized at the round-wire contact region, as shown in Figure 5b. The maximum effective strain remained zero under this loading condition.

At the ERL in the equivalent distributed model, the maximum effective stress at the winding corner was calculated to be 214.4 MPa. As shown in Figure 5c, the maximum stress occurred within the localized contact region between the rigid loading body and the winding corner. The effective strain remained zero under this loading condition, indicating that no plastic deformation occurred in the numerical model at the ERL. Thus, both loading representations predicted an elastic response under their corresponding recommended winding conditions.

The onset of effective strain was examined by progressively increasing the applied load in the equivalent distributed model. The effective strain remained zero until the reaction force reached 141.0 N and first appeared in several elements within the contact region at 144.7 N. This value does not represent an intrinsic yield load of the Fe–Si SMC material but rather the load at which effective strain was first identified under the geometry, contact, and constraint conditions applied in the present numerical model.

At the HVL, the maximum effective stress increased to 876.9 MPa. As shown in Figure 5d, the maximum stress was concentrated at the contact region of the winding corner, as observed under the ERL condition. The maximum effective strain at the HVL was 0.0132 and was localized in the region directly contacting the loading body, as shown in Figure 5e.

After loading to the HVL, the loading body was removed, and the displacement was evaluated when the reaction force reached 0 N. The maximum resultant displacement after unloading was 0.85 μm and occurred within the localized contact region, as shown in Figure 5f.

Overall, no effective strain occurred in either the equivalent distributed model at the ERL or the representative one-turn circular-contact model at $R_{turn}$ = 2.04 N, whereas localized effective strain developed at the contact region under the HVL. After unloading from the HVL, the maximum resultant displacement was 0.85 μm and remained localized within the contact region. These numerical results were subsequently compared with the results of the corner loading tests and optical microscopy observations.

## 4. Experimental Evaluation of Winding-Corner Integrity

### 4.1. Experimental Corner-Loading Response

The ERL and HVL were applied to the winding corners of the actual stator cores, and the force–crosshead displacement responses were recorded during loading. A dedicated fixture was designed and fabricated to maintain a consistent specimen orientation and loading direction. The fixture supported the stator core at an inclination of approximately 45° so that the bisector of the 90° winding corner was aligned with the vertical loading axis of the testing machine. The loading tool connected to the upper crosshead was positioned along the longitudinal direction of the corner contacted by the winding wire, thereby ensuring a consistent load-transfer direction in each test.

The tests were conducted using a 10 kN universal testing machine (AGS-10kNX, Shimadzu Corporation, Kyoto, Japan) at a crosshead speed of 0.1 mm/min [44]. Three stator-core specimens were evaluated under each loading condition. To maintain the same geometric basis for comparison, the force–crosshead displacement response was obtained from the corresponding winding-corner position in each specimen, resulting in three independent measurements per condition. The force and crosshead displacement were continuously recorded during each test. When the measured force reached the prescribed value, the crosshead was returned to its initial position to remove the applied load.

**Figure 6:**
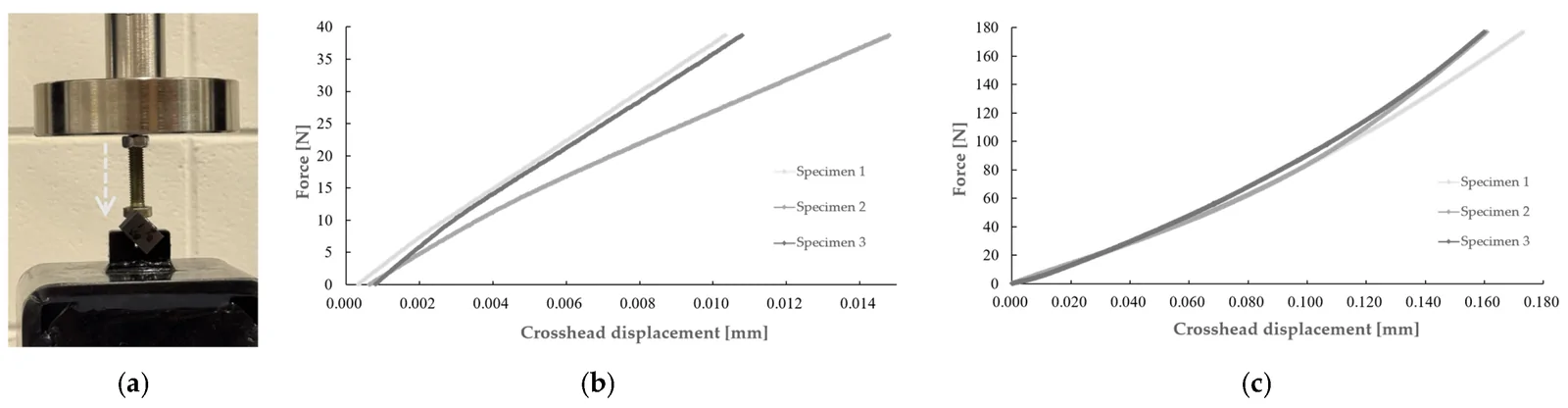
Experimental corner-loading test and force–crosshead displacement responses: (**a**) test setup with the stator core positioned in the fixture, where the arrow indicates the direction of the applied loading, (**b**) representative curves at *Fr* = 38.7 N (ERL), and (**c**) representative curves at *Fu* = 177 N (HVL).

Figure 6 shows the experimental setup and the representative force–crosshead displacement curves obtained under the ERL and HVL conditions. In all curves, the force increased continuously up to the prescribed load without discontinuities indicative of interrupted load transfer. At the first attainment of the prescribed load, the crosshead displacement was 0.0120 ± 0.0025 mm for the ERL condition and 0.1485 ± 0.0184 mm for the HVL condition. These values represent the mean and standard deviation calculated from the three independent stator-core specimens under each loading condition.

The crosshead displacement curves exhibited greater variability under the ERL condition than under the HVL condition. Comparison of the curves in the initial low-load range indicated that this difference primarily occurred during the establishment of contact between the loading tool and the specimen. This relative variability was therefore interpreted as reflecting differences in the initial contact position of the loading tool and the seating condition of the specimen within the fixture.

The measured crosshead displacement reflects the combined compliance of the stator core, loading tool, contact interface, fixture, and testing machine. Accordingly, it was used as a system-level response to quantify the displacement at the prescribed loads and assess the continuity of force transfer across the repeated tests [45,46,47].

### 4.2. Optical Inspection of the Winding Corner Before and After Loading

Optical microscopy was performed to compare the geometry and surface condition of the winding corners before and after the corner-loading tests. The observations were conducted using a stereo microscope (Olympus Corporation, Tokyo, Japan) at a fixed magnification of 20×.

One representative winding corner was selected for each loading condition and imaged before loading. The same corner was observed again after application of the prescribed load and subsequent unloading. The images obtained before and after loading were aligned so that the corner edges had the same orientation. The observation area was centered on the corner with a radius of 0.1 mm contacted by the loading tool. Changes in the edge profile and the occurrence of indentation, cracking, edge chipping, and particle detachment were examined.

Fine burrs remaining at the winding corners after compaction of the stator cores were removed before testing. The initial observations at 20× magnification confirmed that the rounded corner was continuously formed along the longitudinal direction. The edge contacted by the loading tool also exhibited a smooth and continuous profile without distinct geometric discontinuities or localized edge defects. This initial corner geometry was therefore used as the reference for the subsequent before-and-after comparisons.

**Figure 7:**
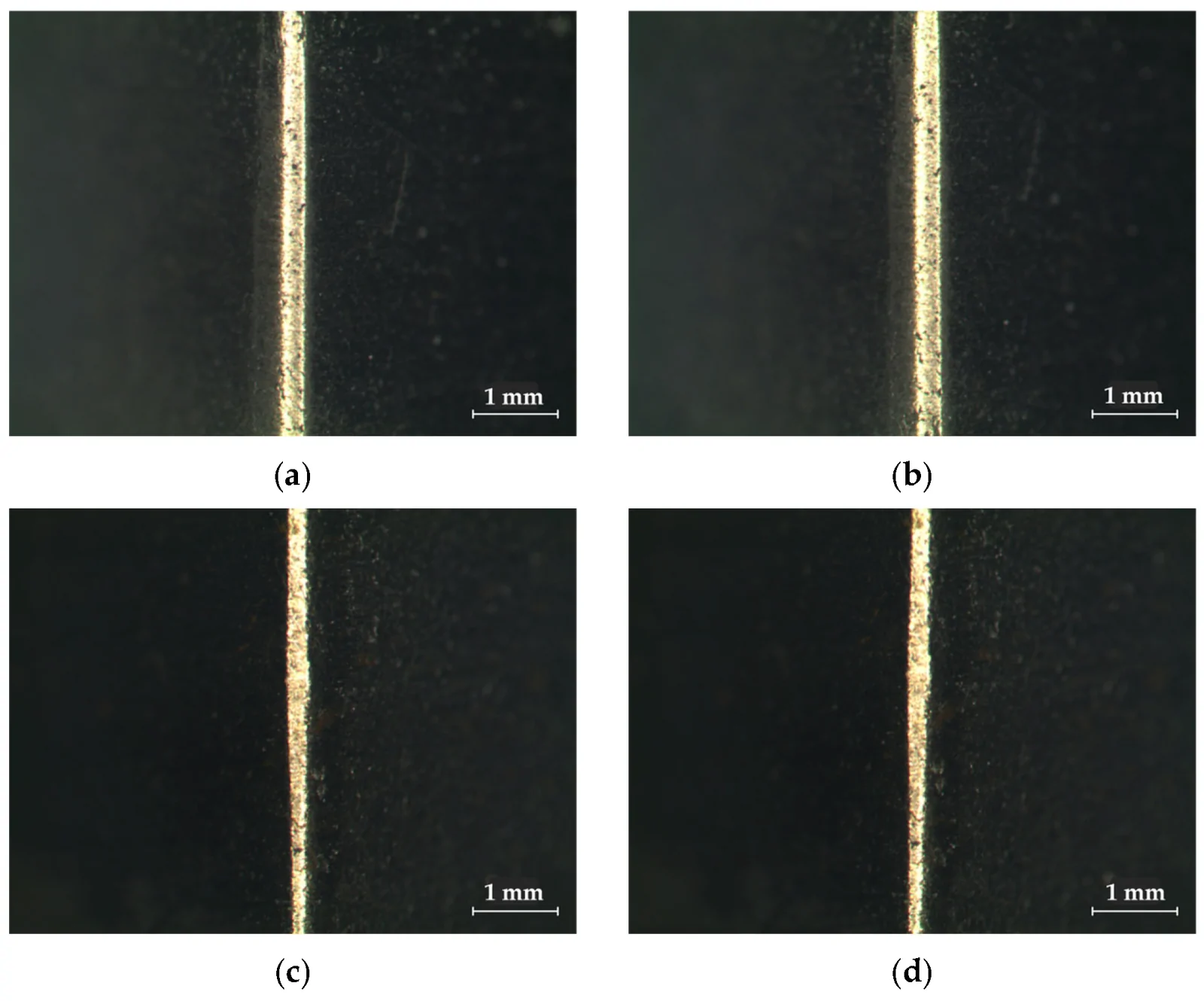
Optical micrographs of representative winding corners before and after the corner-loading tests: (**a**) before loading and (**b**) after unloading at *Fr* = 38.7 N (ERL); (**c**) before loading and (**d**) after unloading at *Fu* = 177 N (HVL). All images were acquired at a fixed magnification of 20×.

Figure 7a,b show the winding corner before application of the ERL and after unloading, respectively. The position and continuous geometry of the edge were maintained between the two images, and no indentation or profile change was detectable in the region contacted by the loading tool at 20× magnification. Cracking, edge chipping, and particle detachment were also not detected.

Figure 7c,d show the winding corner before application of the HVL and after unloading, respectively. The rounded edge remained continuous along the longitudinal direction after unloading. No cracking, edge chipping, indentation, or particle detachment was detectable in the contact region at 20× magnification.

The optical microscopy results showed no change in the edge profile or surface condition detectable at 20× magnification after unloading under either loading condition. At the HVL, the numerical analysis predicted localized effective strain and a maximum resultant displacement of 0.85 μm after unloading, confined to a small region at the direct contact interface. No corresponding visible change in the edge profile or surface condition was identified in the 20× optical images. Therefore, the optical results were interpreted as showing no surface damage detectable at the applied magnification.

### 4.3. Integrated Numerical and Experimental Evaluation

The finite element results presented in Section 3 were integrated with the experimental results presented in Section 4.1 and Section 4.2 to evaluate the load range applied to the winding corner of the stator core. The evaluated load range extended from the ERL to the HVL. The load ratio, *λ*, was normalized by the ERL, and the verification range evaluated in this study was expressed as follows:

(2) $$F_{r}\leq F\leq F_{u}$$

(3) $$1.00\leq \lambda =\frac{F}{F_{r}}\leq \frac{F_{u}}{F_{r}}=4.57$$

where *F* is the verification load applied to the winding corner. Equations (2) and (3) define the range over which the deformation and surface condition of the stator-core corner were evaluated, extending from the ERL to the HVL.

In the representative one-turn circular-contact model, the per-turn resultant load of $R_{turn}$ = 2.04 N produced a maximum effective stress of 22.9 MPa, while the effective strain remained zero. In the equivalent distributed model at the ERL, the loading-body displacement was 1.15 μm and the maximum effective stress was 214 MPa, while the effective strain remained zero. Thus, both loading representations predicted an elastic response under their corresponding recommended winding conditions. Optical microscopy of the stator-core winding corner subjected to the ERL showed no edge-profile change, indentation, cracking, edge chipping, or particle detachment detectable at 20× magnification after unloading.

During progressive loading, the effective strain remained zero up to 141.0 N and first appeared in several corner elements contacting the loading body at 144.7 N. These loads correspond to *λ* = 3.64 and *λ* = 3.74, respectively. The elements exhibiting effective strain were concentrated within the corner region directly contacting the loading body, and the affected region did not extend throughout the core.

The HVL corresponds to *λ* = 4.57. Under this condition, the maximum effective stress and maximum effective strain were 876.9 MPa and 0.0132, respectively. After unloading, the maximum resultant displacement was 0.85 μm, while the Z-direction displacement ranged from −0.834 to 0.402 μm. These values represent the maximum nodal displacements within the localized contact region and do not correspond to the overall indentation depth of the winding corner.

In the corner-loading tests of the actual stator cores, the force increased continuously up to the ERL and HVL without an abrupt force drop during loading. The winding corner observed after loading to the HVL and subsequent unloading retained a continuous edge and rounded profile. No indentation, cracking, edge chipping, or particle detachment was identified at 20× magnification within the region contacted by the loading body.

Comparison of the numerical and experimental results showed that the response remained elastic at the ERL, and no identifiable change in the corner geometry was observed after unloading. At the HVL, the numerical analysis predicted a maximum effective strain of 0.0132 and a maximum resultant displacement of 0.85 μm within the local contact region. Optical microscopy at 20× magnification showed no identifiable geometric change or surface damage at the winding corner after unloading.

The continuum model represents the macroscopic constitutive response of the processed SMC. Particle-scale heterogeneity, residual pores, and insulating interfaces may influence the local redistribution of stress and strain within the contact region.

## 5. Conclusions

This study evaluated the local mechanical response of an Fe–5.0 wt.%Si SMC stator-core winding corner under loading conditions associated with direct winding. Finite element analysis, component-level corner-loading tests, and optical microscopy were combined to assess the local deformation and geometric integrity of the winding corner with a nominal radius of 0.1 mm.

The numerical analysis showed an elastic response to the ERL, whereas localized effective strain developed under the HVL. At the HVL, the maximum effective strain was 0.0132, and the maximum resultant displacement after unloading was 0.85 μm, with the deformation concentrated in the local contact region. In the component-level tests, the prescribed loads were applied without an abrupt force drop, and optical observations at 20× magnification showed no identifiable indentation, cracking, edge chipping, particle detachment, or change in the corner profile after unloading. These results characterize the local mechanical response of the investigated Fe–5.0 wt.%Si SMC winding corner for the combination of manufacturing process, target density, insulation treatment, corner geometry, wire specification, and loading conditions examined in this study.

Further studies will extend the present evaluation to stator cores with different slot-opening geometries, tooth geometries, corner radii, and cross-sectional dimensions to examine their effects on local contact stress and residual deformation. These investigations will support the development of design criteria for maintaining winding-corner integrity in direct-winding SMC stator cores.

## Figures and Tables

**Table 1:** Fe-5.0 wt.%Si raw powder chemical composition and average particle size.

Powder	Element	Content [wt.%]
Fe-5.0 wt.%Si	Fe	94.85
	Si	5.02
	O	0.13

**Table 2:** Bulk mechanical properties of the Fe–Si SMC.

Property	n	Mean ± SD	CV [%]
Compressive strength [MPa]	5	1226.4 ± 22.0	1.79
Vickers hardness [HV1]	5	358.2 ± 6.1	1.70

**Table 3:** Nominal 0.2% proof stress and proof load of the AI-EIW wire specimens.

Specimen	*σ*_0.2_ [MPa]	*F*_0.2_ [N]
S1	177.84	201.14
S2	152.65	172.65
S3	147.79	167.11
S4	157.24	177.84
S5	146.83	166.03
Mean ± SD	156.47 ± 12.66	176.95 ± 14.32

## Data Availability

The original contributions presented in this study are included in the article. Further inquiries can be directed to the corresponding author.
